# 2294. Severity and characteristics of patients hospitalized during Delta and Omicron-predominant COVID-19 time periods in Los Angeles County

**DOI:** 10.1093/ofid/ofad500.1916

**Published:** 2023-11-27

**Authors:** Carmon Greene, Heidi D Sato, Kelsey OYong, Elizabeth Traub, Zachary Rubin

**Affiliations:** Los Angeles County Department of Health, Los Angeles, California; Los Angeles County Department of Public Health, Los Angeles, California; Los Angeles County Department of Public Health, Los Angeles, California; Los Angeles County Department of Public Health, Los Angeles, California; Los Angeles County Department of Public Health, Los Angeles, California

## Abstract

**Background:**

We examined risk factors and disease severity in patients hospitalized during Delta and Omicron SARS COV-2-predominant periods in a diverse metro area.

**Methods:**

A retrospective cohort study was conducted using data reported to Los Angeles County Department of Public Health on adults aged 18 years and older with a laboratory-confirmed COVID (PCR/NAAT) test collected on or within 14 days of hospital admission between 6/19/2021 - 1/3/2023. Case data were matched with: 1) California Immunization Registry data to determine vaccination status and 2) whole genome sequencing results when available.

Infection with an inferred variant was defined as having a laboratory collection date during the time period when the variant exceeded 50% of sequenced specimens (Delta-predominant period: 6/19/2021-12/11/2021; Omicron-predominant period: 12/18/2021-1/3/2023). Confirmed infection was defined as having a sequenced specimen with either Delta or Omicron variant. Demographic and clinical metrics were examined (Tables 1-4). Patients with Delta and Omicron were compared using Chi-square tests. P-values < 0.05 were considered statistically significant.

**Figure:**
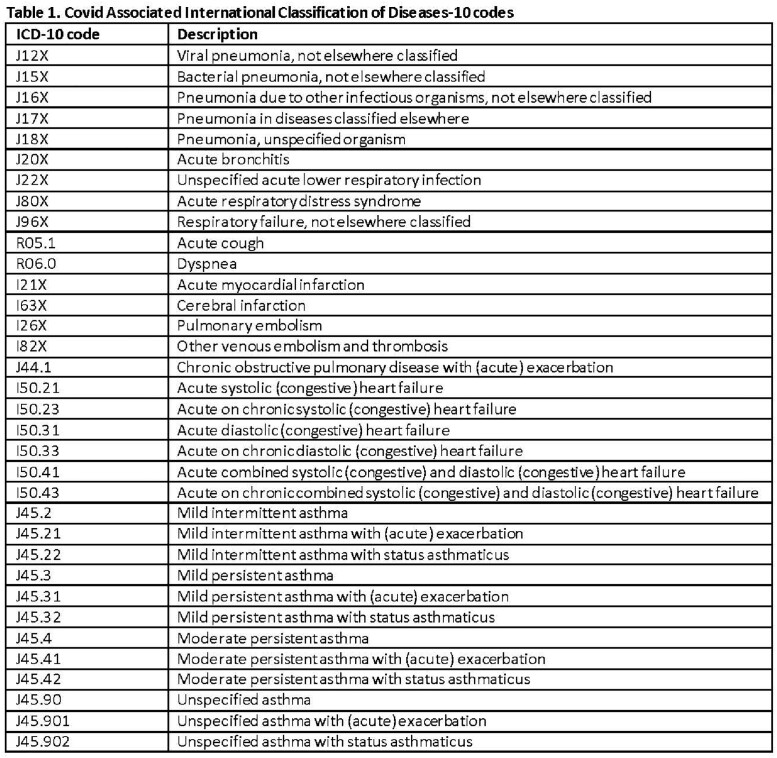

**Figure:**
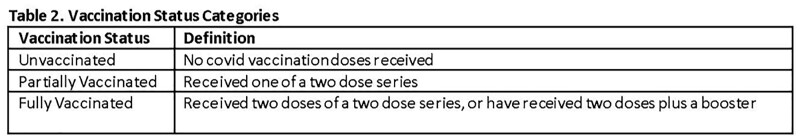

**Results:**

During the study period, 13,790 inferred Delta and 38,264 inferred Omicron hospitalized cases were reported. 2,660 cases (of 61,983 sequenced specimens) had confirmed Delta and 1,983 (of 61,534 sequenced specimens) had confirmed Omicron infections. Cases hospitalized with both inferred and confirmed Delta were more likely to have a COVID-related hospitalization, admitted to ICU and longer length of stay (Tables 3, 4). Cases hospitalized with both inferred and confirmed Omicron were significantly older and more likely to have a comorbidity (Tables 3, 4).

**Figure:**
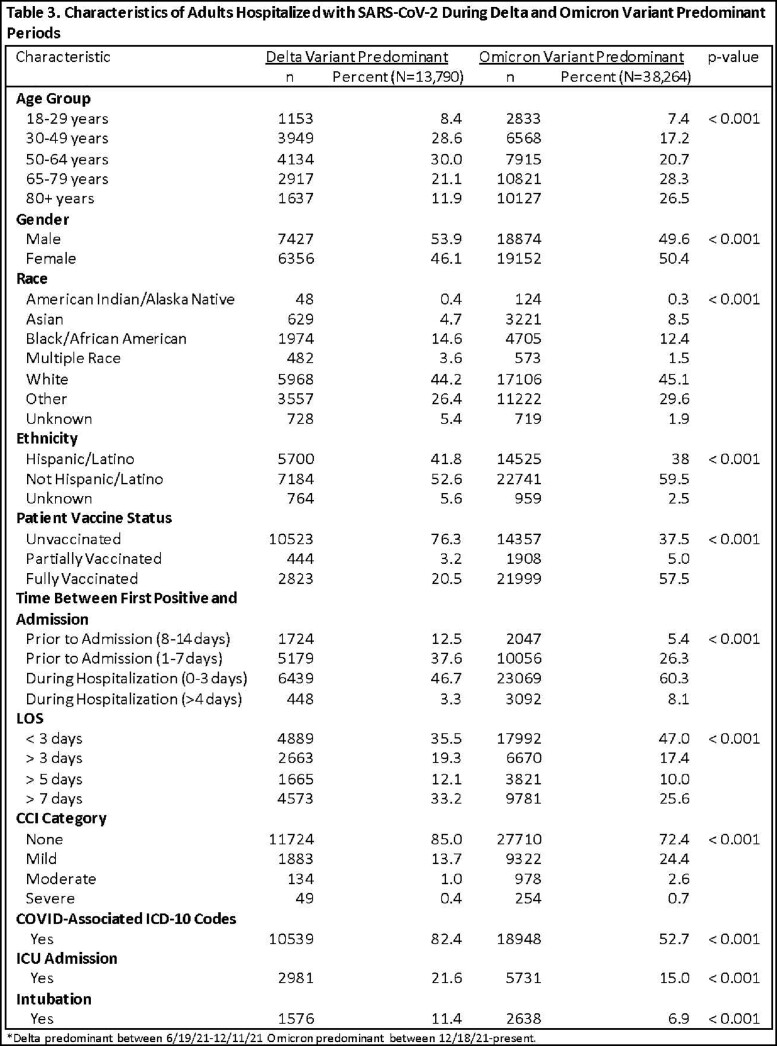

**Figure:**
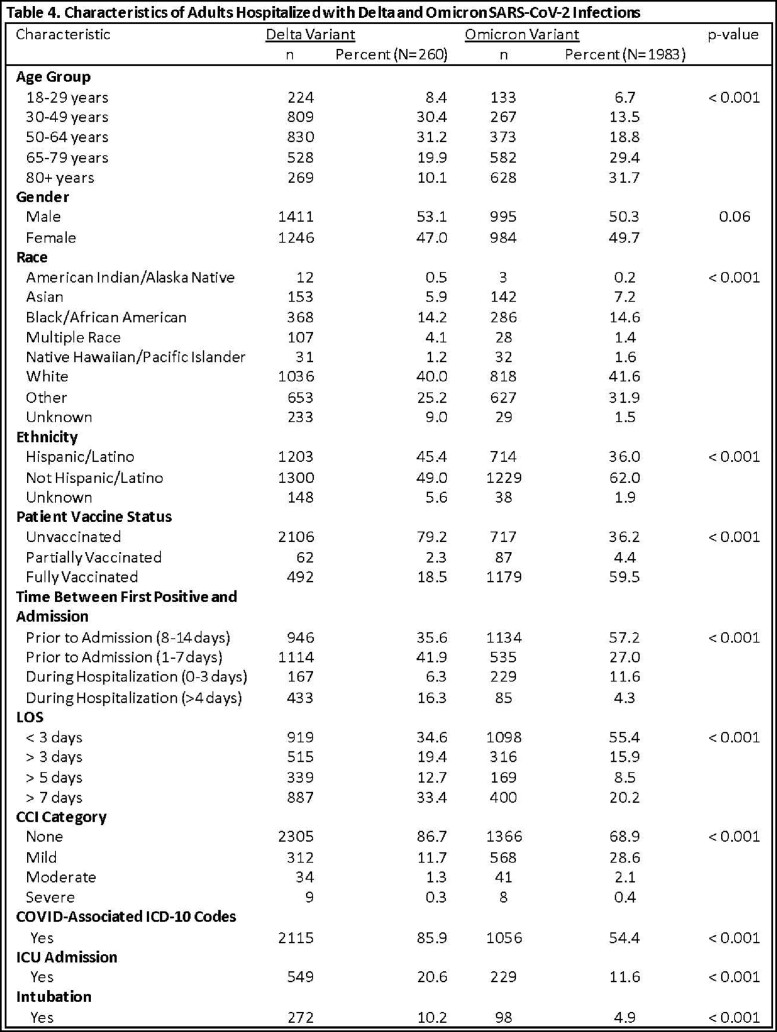

**Conclusion:**

Based on the longer length of stay and higher ICU admission, COVID patients hospitalized during the Delta-predominant time period have a more severe outcome and are more likely to have COVID-related diagnoses than COVID patients hospitalized during the Omicron-predominant time period.

This study was limited by an unadjusted analysis, which did not control for potential confounders during the different time periods, such as vaccination coverage, prior infections, hospital capacity, and percent of sequenced specimens.

**Disclosures:**

**All Authors**: No reported disclosures

